# Reading Between the Lines: A Pursuit of Estimating the Population Prevalence of Mental Illness Using Multiple Data Sources

**DOI:** 10.1177/07067437211016255

**Published:** 2021-05-10

**Authors:** Jordan Edwards, Katholiki Georgiades

**Affiliations:** 1 Offord Centre for Child Studies, 3710 McMaster University, Hamilton, Ontario, Canada

**Keywords:** epidemiology, prevalence, mental disorders, Bayesian statistics, triangulation, evidence synthesis, mental health service planning, mental health policy

## Abstract

Population-based prevalence estimates of mental illness are foundational to health service planning, strategic resource allocation, and the development and evaluation of public mental health policy. Generating valid, reliable, and context-specific population-level estimates is of utmost importance and can be achieved by combining various data sources. This pursuit benefits from the right combination of theory, applied statistics, and the conceptualization of available data sources as a collective rather than in isolation. We believe there is a need to read between the lines as theory, methodology, and context (i.e., strengths and limitations) are what determines the meaningfulness of a combined prevalence estimate. Currently lacking is a gold standard approach to combining estimates from multiple data sources. Here, we compare and contrast various approaches to combining data and introduce an idea that leverages the strengths of pre-existing individually linked population-based survey and health administrative data sources currently available in Canada.

As psychiatric epidemiologists interested in producing population-based estimates of mental illness, we must be guided by the strengths and limitations of the data we use. The pursuit of estimating prevalence by combining multiple sources of evidence benefits from the right combination of theory and applied statistics. Important questions remain, however, on how best to conceptualize and combine data arising from different sources, such as survey and health administrative data, to arrive at an accurate estimate of mental illness in the population. Recently, there have been a number of suggested approaches, including both a Bayesian and now a triangulation approach from Vigo et al., which have helped to move this discussion forward.^1,2^

One of the greatest challenges of this work is establishing common measurement and determining whether various data sources are measuring the same underlying construct. One method for testing this is to use linked data to measure concordance across sources. Canada is positioned to tackle this challenge, as the number of provincial and national population-level data linkages has increased. Recent Canadian findings using linked population-level survey and health administrative data revealed that while both sources of data provided comparable prevalence estimates of mood or anxiety disorders, there was high discordance between these data sources.^3^ As such, we now know that these distinct data sources are either capturing different people with a mood or anxiety disorder or the same group of people at different stages of their mental health trajectory.^3^ To fully capitalize on the strengths of various data sources, researchers must conceptualize available sources as a collective rather than a series of sources in isolation. The idea that the strength of one data source may be its ability to reduce bias in another is important. Combining evidence involves balancing estimate precision, which can be increased by maximizing available evidence, with estimate accuracy (i.e., validity), which involves quantifying and when possible reducing estimated biases from various data sources. While not mutually exclusive, this balance often influences methodological considerations.

In the pursuit of estimating mental illness in the population using multiple data sources, there is a need to *read between the lines* as theory, methodology, and context (i.e., strengths and limitations) are what determines the meaningfulness of a combined prevalence estimate. Here, we compare and contrast various approaches to combining data, and we introduce an idea that leverages the strengths of pre-existing individually linked population-based survey and health administrative data sources currently available in Canada.

## Methodology for Combining Estimates from Multiple Data Sources

### Triangulation Approach

Vigo and colleagues introduced an approach to combining estimates from many different data sources using meta-analysis and triangulation.^1^ This approach offers the ability to provide timely estimates that are informed from various Canadian and international data sources, including evidence from survey and health administrative data. Furthermore, the authors introduce the idea of contextualizing estimates of mental illness by disorder class, age, sex, and presenting severity fractions that are informed by the Global Burden of Disease framework.^4^ This approach accompanied with the proposed framework for estimating service needs based on the findings from their triangulation analysis, positions this work as a forward-thinking approach to using multiple data sources to estimate mental illness in the population.

There are a number of key assumptions that need to be met for the triangulation approach to improve the validity of an estimate. These include (1) various data sources included in the model are measuring the same underlying construct and (2) the various data sources that are being combined have differing and unrelated key sources of potential biases.^5^ We believe these assumptions might compromise the validity of estimates drawn from a triangulation approach. First, evidence suggests that various data sources (i.e., survey data, administrative data) may be measuring different components of the same underlying construct rather than the exact same outcome.^3^ Second, enumerating the magnitude, direction, and independency of biases between and within various data sources is a challenge. Finally, while paired with a meta-analysis, the triangulation approach maximizes available evidence, thus increasing the precision of a combined estimate, accuracy (i.e., validity) may be compromised. Combining sources that provide greater homogeneity in the assessment of the underlying construct may provide more accurate estimates.

### Bayesian Approach

An alternative approach to triangulation, which is currently being used in the global burden of disease study,^6^ and to forecast mental health service needs in the UK,^7^ is a Bayesian analytical framework. One of the major strengths of this methodology is its ability to model an outcome using not only the prevalence estimates from various sources, but prior information which can be integrated to enhance our confidence in estimates drawn from various sources. This is important when combining data sources which measure different components of the same outcome. In these cases, a Bayesian model can integrate prior information that characterize the relationship between various data sources (i.e., their concordance, observed or estimated) as well as prior information that can help guide our confidence in both measures compared to a gold standard measure. This was shown in a recent Bayesian analysis conducted in Ontario.^2^ While this approach may be used to combine estimates with varying potential biases, one of the strengths of this approach is its flexibility in using prior information to adjust the model to reduce known biases. This has important implications for estimating mental illness among Canada’s diverse population, where various measures may provide more valid estimates among certain sub-populations. We believe there are two major strengths of the Bayesian approach. First, is its ability to produce combined estimates that are informed from prior information, which allow researchers to leverage the strengths of various data sources while incorporating and adjusting for potential biases to enhance the accuracy of pooled estimates. Second, it has the distinct advantage of forward uncertainty propagation through its pairing with simulation-based methodologies.^8^ This allows for the estimation of uncertainty surrounding individual prior estimates and combined estimates. This not only provides insight into our confidence in a combined estimate but also into the quality of the evidence that is available to inform the model. Furthermore, Bayesian modelling can be used to measure mental illness over space, time, and by sub-population.^9^ As such, we believe this approach is well suited for measuring mental illness at the population level.

One of the challenges of using a Bayesian analysis is the time required to create an initial analytical infrastructure. Once established, however, new data can be added in an iterative fashion, which would provide an opportunity for timely dissemination. A Bayesian approach may also require extensive collaboration to inform the model when prior information is unavailable. As such, this approach presents the opportunity to integrate the values and perspectives of a diverse range of researchers and knowledge users.

## Measuring Mental Illness in the Population

While the technique used to combine data is important, we believe developing our theory of measurement is equally important. The field has room for development in this area. For example, there may be benefits to moving away from focusing on measuring one single overall prevalence to a more contextualized series of prevalence estimates, including estimates of population need, service use, and unmet need. This would provide opportunities to inform service planning, and evaluate service targeting for various mental disorders. To that end, we believe individually linked population-based survey and health administrative data can support these efforts. Specifically, by leveraging (1) the ability of surveys to estimate community need and community mental health service contacts; (2) administrative data’s ability to estimate physician-based service contacts; and (3) the linkage of both data sources to measure the discordance between indicators of mental health needs and service use to estimate unmet need in the population. Fully capitalizing on currently available data linkages in Canada may reveal new ways of measuring mental illness in the population, which may better serve policy makers.

## Reading Between the Lines

The production of valid, reliable, and context-specific population-based estimates of mental illness are the foundation of health service planning and strategic resource allocation. These estimates are used to inform and evaluate equitable public mental health policy initiatives and the performance of our mental health care system. We believe in the idea of combining various data sources for improving our ability to generate reliable estimates of mental illness, including estimates of population need, service use, and unmet need. Navigating the data landscape and identifying, quantifying, and leveraging the strengths of various data sources (i.e., and adjusting for their limitations), in a way that improves these estimates is our challenge. We believe a Bayesian approach will allow us to *read between the lines* and fully capitalize on the strengths of various data sources available in Canada. Nevertheless, there is currently no gold standard approach to combining estimates from multiple data sources. As such, we must work to compare and contrast various approaches, including the triangulation approach introduced by Vigo and colleagues, which has moved this field forward.^1^
